# On the Application of Tincture of Iodine as an Ectrotic Remedy in Small Pox

**Published:** 1853-12

**Authors:** James Crawford

**Affiliations:** Professor of Clinical Medicine, M‘Gill College, and Physician to the Montreal General Hospital


### RECORD OF MEDICAL SCIENCE.

### PATHOLOGY AND PRACTICE OF MEDICINE.

*On the application of Tincture of Iodine as an ectrotic remedy in Small Pox.* By James Crawford, M.D., *Professor of Clinical Medicine, M'Gill College, and Physician to the Montreal General Hospital.*

It is now upwards of nine years since I first recommended the application of tincture of iodine as an ectrotic remedy in small pox, and although I observe that the suggestion has been noticed by Dr. Copland, in his Dictionary of Medicine, and by Dr. Dunglison, in his work on Therapeutics, and also by some others in the United States, I would nevertheless desire again to draw the attention of the medical profession to the benefit that a more extended experience has convinced me would follow a general application of the remedy.

Epidemics of the dangerous malady of small pox, have been fortunately rare amongst us, and therefore the opportunity of further testing this remedy had not been afforded me, till the latter end of the last year, and earlier months of the present, during which period I have had occasion to treat, both in hospital and private practice, several cases of very grave variolous disease, and would desire to lay my further experience before the profession, anxious that a fair trial and just estimate of the application should be made, while I feel fully confident that it will maintain the reputation I have deemed it deserving of. I would here appeal to those who have seen much of the natural small pox, or its effects, how few cases escape pitting and unseemly scars, when the disease is allowed to run its course without interference, and I would ask, how many attempts have been made in consequence, to supply an ectrotic remedy, and how difficult of application, or disagreeable, and even inefficacious, are any that have been hitherto recommended. The Herculean undertaking of cauterizing the several individual pustules, in severe cases, quite precludes its application. I have reason to think the compound tincture of iodine, a very powerful and efficacious remedy, which has been tried with very satisfactory results in the Montreal General Hospital, by my friend Dr. Campbell, but from my being under the impression that the addition of the hydriodate of potass caused more pain, I have not employed this form. The disagreeable mercurial mask, the inefficacious covering of gold leaf, cotton, or collodion, are now in a great measure laid aside. I stated formerly, on the occasion of my first suggestion of this application, in the Medical Gazette published in this city in 1844, that I was led to try it in small pox from the very marked benefit I had derived from its use in erysipelas, and various other cutaneous diseases, for several years previously. I was then satisfied of its *anti-phlogistic* powers and *soothing* effects, and trusted that a more general employment of it in variola would establish its claims to general confidence.

During the late epidemic of variola, I have had several opportunties of trying its powers, and my cases have been observed by many members of the profession, to whom the issue has afforded every satisfaction. I have reason also to know, that several medical practitioners have followed my example with success, while others have made only a very *imperfect* and *insufficient occasional* application, which neither could afford a satisfactory result, nor determine the advantages derivable from it.

I have been favored with the opinion of several physicians of this city, of the highest standing in the profession, on the advantage of using this remedy, which I subjoin.

The application I have used is a saturated solution of iodine, in spirit of wine, *which is to be brushed freely over the face once or twice daily, from the earliest day of the eruption that is practicable, and continuing the repetition of the application daily, or oftener, during the period of the maturation of the pustules. The earlier the application is commenced the more efficacious it proves. The inflammatory and ulcerative processes are controlled, and the intolerable itching relieved,* by which means *scratching,* and its evil consequences, are obviated. For some time I was disposed to confine the application to the face, as being the part most disposed to ulceration and pitting, as well as that most desirable to be preserved from marks. I have, however, on many occasions applied it to various other parts, for the sake of experiment, or contrast, and also to relieve the intolerable pruritus, and have even extended it over nearly the whole body, at the patient's desire, without any evil consequence or inconvenience from the most extended application. The relief it affords to the itching, (if it conferred no other boon), would of itself be a sufficient recommendation of the application. Its antiphlogistic and febrifuge properties, however, are very manifest, and I have no doubt *modify* and *moderate* the *fever*, and thereby operate in a most salutary manner. The medical treatment I have combined with it is so simple and mild, that a great deal cannot be attributed to it; being merely small doses of calomel and Dover's powder occasionally, during the day, and night, as a sedative. When pits are left, I have observed that they principally occur on the nose, and I am inclined to think that this may in some degree be owing to the insufficiency of the application to this sensitive part, or from the disagreeable vapor causing irritation of the schneiderian membrane, or eyes, which makes the patient more desirous to escape from its application; but even this inconvenience may be easily obviated, by keeping the eyes shut, and, if requisite, stopping the nostrils.

The immediate effect of the application is pain, which is more complained of by some than by others. It speedily subsides, and gradually abates in severity, after the first few applications; and the relief to the itching it affords is so gratifying to the patient, and the effects so manifest to the friends, that they always remark the contrast of the parts "painted," with those left "unpainted;" and frequently request a further extension of the application. I will record very briefly, a few of the cases, some of which were treated in the Montreal General Hospital, and some in my private practice:—

Case 1.—*Variola Discreta.*—J. H., aged 19, admitted into the Montreal General Hospital on the 31st October, 1852, under my care, on the third day of a variolous eruption, which covered the face and limbs very profusely, although distinct. The face was swelled, the tongue covered by a pustular eruption, and there was salivation. The initiatory fever had been severe. He complained much of the itchiness of the face, his pulse was full and frequent. He was ordered to be painted over the face, and to have small doses of calomel and Dover's powder 3 times a-day. One of his arms was also painted for the object of comparison with the other. These parts soon exhibited a marked contrast from those left unpainted. The pustules remained small and formed thin scabs, which fell off early, and left the subjacent parts free from pits. Although this case was grave, there was no bad symptom. It was seen by several medical gentlemen, who expressed their satisfation and conviction of the beneficial effects of iodine, and that it had prevented pitting and marks. Some weeks after convalescence, his face was quite smooth. The patient was uncertain whether he had been previously vaccinated.

Case 2.—*Semi-confluent Small Pox.*—E. B., aged 19, admitted into the Montreal General Hospital, 30th November, 1852, on the second day of variolous eruption, which had been preceded by high fever, vomiting, epigastric and lumbar pains. It was copious on the face, which was much swelled, his spirits good, slept tolerably well, and without delirium. Eruption became very profuse over the limbs, and confluent on some parts, areola bright. The fever was moderate. Ordered to be painted with tincture of iodine, and to have the calomel and Dover. The crust formed a complete mask over the face, but was thin. The buccal mucous membrane did not appear to be involved in the eruption. The application was made daily, and the case progressed favorably. There were no depressions or marks left on the face. The iodine was also applied to different parts of the body to relieve the itching with very satisfactory results. Did not know whether he had been previously vaccinated.

Case 3.—*Semi-confluent.*—M. M. ætat. 7, (private patient,) to whom I was called on the second day of eruption, which had been preceded by smart fever and vomiting. The eruption was profuse, but distinct, over the face and extremities, with few pustules on the body, the mucous membrane free from it. The patient was said to have been vaccinated. She was ordered, as usual, to be painted. She did not make much complaint of the application, although of such tender years. The pustules on the painted part remained small and flat, quite unlike the other parts. Several pustules on the limbs became confluent—the areola rosy. On the 9th day, some of the pustules on the limbs had a hemorrhagic appearance, the scabs on the face were thin, and the secondary fever light. There was little swelling of the face, and no salivation. About the 10th day, she was attacked by rheumatic pains and swelling of the ankles and knees. Her bowels became disturbed for some days; an abscess formed at the ankles, elbow and axilla; these were discharged. Her strength was supported from an early period by nutritious diet—beef tea, arrow root, wine and quinine. About the 21st day of the eruption, she began to cough, accompanied by a mucous rale, and profuse expectoration, with great dyspnœa at intervals. An emetic, a sinapism, and pediluvium relieved her for the time. The mucous secretion continued profuse, and her strength failed, and she sank on the 31st day. There were only a few small *superficial* pits on her face, which would not have been very observable had she lived. The irritation from the rheumatism, and the discharge from the several abscesses, together with the profuse tenacious bronchial discharge, and consequent orthopnea, carried her off. The case would otherwise have been very satisfactory, although the application was commenced later than was desirable.

Case 4.—*Semi-confluent Variola.*—Miss E. M., aged about 18, (private patient,) after a smart fever, accompanied by severe epigastric pain, intolerance of light, redness of the conjunctiva, and slight sore throat, an eruption of papulæ appeared on the face and wrists. I saw her on the second day of eruption, when it was thickly out over the face and limbs. It soon spread over the whole body, and was very profuse, but kept distinct, except at a few parts. The iodine was applied to the face and back, with so much relief to the patient, from the itching, that she on many occasions made her sister apply it to various parts; in fact I

might say it was used all over her body, which circumstance she confessed after her convalescence. The secondary fever was severe, accompanied by much gastric irritation. There was also a good deal of suffering from a rheumatic affection of her wrists and ankles, which rendered her very helpless. She, however, got well, without any further troublesome symptom. The scabs were thick, and remained a long time on the right side of her nose, which (as was remarked by her sister) had been less assiduously painted, from that side being turned to the wall, and inconvenient to get at. On this part there remain several small superficial depressions, and the forehead has some very slight marks, only discernible *en profile*, which I expect will not be perceptible in a little time. The face is pale and without any stain, and generally quite smooth. The case was very severe and was seen by several medical gentlemen, who expressed their satisfaction of the efficacy of the remedy. It is doubtful if this patient went through regular vaccinia.

Case 5.—R. C., ætat. 15. I was called to this case on the 5th day of the eruption. The girl had been under the care of a medical practitioner, who had not applied the iodine, although it was suggested to him by the priest, who had seen its advantages in the previous case. The eruption over the face was flat and ill-filled. Although profuse, it was distinct over the body. She was a delicate, dwarfish girl, subject to splenitis. At the period I saw her, she was very weak, depressed in spirits, and sleepless. She was ordered a small quantity of wine and water, and beef tea frequently, calomel and Dover's powder, and to have the face painted. Although the expectation of benefit was much lessened, by the late period of the application, it caused, as usual, some pain, but at the same time afforded so much relief from the itching, that she frequently desired its reapplication. The eruption became confluent on several patches on the limbs ; but little eruption on the body. The face swelled, and there was salivation. The scab on the face formed a complete mask, but not very thick. Her spirits revived, and her strength was maintained by wine and soups. Her feet, legs and wrists became painful and swelled. She, however, recovered well in about three weeks. There remain several small superficial pits on the face, which could not well be otherwise, as the application was so late in being applied, and a mark of a scratch she made before the iodine was applied. But they are evidently very much modified even by the late use of the remedy, and the relief to the itching derived from it was manifest, from her often desiring its application and extension over other parts. Several boils took place on different parts, but she soon recovered. This patient had never been vaccinated. Her eldest sister was vaccinated during the progress of the case, and passed through the stages, in a satisfactory manner.

Case 6.—*Variola confluenta.*—A. A., ætat. 15, a delicate looking boy, had never been vaccinated, nor any of his family, three of whom were vaccinated on the occasion of my being called to see him, and all passed through the regular stages in a satisfactory manner. This boy had, a short time before his illness, received a visit from a young friend, just recovered from an attack of variola. The primary fever and epigas tric pain were pretty severe. The eruption was profuse over his face and extremities when I saw him on the second day. The iodine was applied in an unsatisfactory manner, from the interference of the patient and his mother. The eruption soon became very profuse, and confluent on many parts. The tongue and fauces were covered by ulcers; the voice scarcely audible; some cough and expectoration. The iodine produced such a soothing and satisfactory effect, that he soon desired its re-application, and it was extended to various parts to relieve the itching. The case, although very severe, went on well. Secondary fever was high, and there was much distress from the mucous membrane of the larynx, and from the pustules on the scrotum, and pains of his hands and soles of his feet, which are covered with pustules. He also suffered from rheumatism of the ankles and wrists, which were much swollen. The Dover and calomel afforded him relief and sleep at night. Beef tea and arrow root were ordered from the earliest day, and latterly wine and quinine. He was convalescent in three weeks, and able to sit up, in good spirits, saying he could dance with nurse, if the sores on his feet did not prevent him. Scarcely a trace of pit or depression being left on the face, whilst the parts unpainted showed numerous pits. On the 23d day from the appearance of the variolous eruption, an erysipelatous blush appeared on the forehead, and a similar one on the knee. An abscess formed in the axilla, and also on the eyelid and ankle. His back also became painful, and affected by erysipelas, and a smart fever supervened. His bowels discharged large quantities of ochrey looking fermenting and very offensive evacuations, for three or four days, when the fever and erysipelas subsided. About the 30th day the fever returned and assumed a typhoid type; dark, black, dry tongue; muttering delirium, subsultus tendinum, &c. &c. He continued in this precarious state for a week, when he became quite intellectual, and able to tell his wants, and good hopes were entertained of his recovery, when suddenly, after two days of this favorable state, he was seized with dyspnœa and hurried breathing, and died in a few hours. The treatment is omitted, as not being an object on the present occasion. The most satisfactory results were observed to attend the use of the iodine, both by allaying the irritation and preventing marks, scarcely any being perceptible. This case was seen by Dr. Campbell, in consultation, and by others, to witness the effects of the remedy.

I have treated several other slighter cases, in which the iodine was used in all with marked benefit in relieving the itching, and in all probability preventing pitting, as even in cases where the eruption is sparse, pitting may follow. I have also seen several severe cases in which it was tried in the Montreal General Hospital, under the care of other physicians, with the most satisfactory results, a summary of which accompanies this notice.

I think I may add without overrating the advantages of the application, that being a powerful antiphlogistic, while it lowers the inflammatory action, it thereby controls the general fever, and moderates the risk and mortality from the secondary fever.

The two fatal cases which I had during the present epidemic, being evidently rendered so by other causes than variola, namely, in one, by erysipelas and typhus fever, supervening during convalescence, on the thirty-first day after the appearance of the variolous eruption. The other fatal case was carried off on the thirtieth day by continued irritation, and wasting from rheumatism, abscess and bronchitis, with profuse mucous discharge.

I have very great pleasure and satisfaction in adding the testimony of Dr. Bergin, of Cornwall, to the beneficial effects of iodine in small pox; who had in 1849 an opportunity of using it on a very extended scale, such as rarely is the lot of any individual in this country. The following summary, which is founded on returns made to the Hon. Colonel Bruce, Superintendent General of Indian affairs, is very brief, but it comprehends all that can be desired in support of the claim of this application, as an ectrotic remedy. Dr. B. had witnessed the early experiments I had made on this subject, during his pupilage in Montreal, and gladly availed himself of the unusual opportunity he had, when employed by the Colonial Government, to afford his professional aid, to a tribe of Iroquois Indians at St. Regis, on the banks of the St. Lawrence.

He briefly states, "I have treated 300 cases of small pox among the Iroquois Indians at St. Regis, during an epidemic in 1849. Of these 200 were very severe, either confluent or partially so, and to whom iodine was applied, as follows:—The whole face was painted, daily from the earliest day that it could be done in *eighty-five cases* of confluent, or semi-confluent small pox, out of which *only seven exhibited any marks, and these were slight. Half the face* was painted in *seventy* cases of grave disease; of these, *sixty-one were free from any marks on the painted side*, five were badly pitted, and four slightly, on the painted side, while the *unpainted* side had numerous marks and pits, exhibiting a very striking and marked contrast, fifty cases were painted at different periods, during the maturation of the pustules, upon which the tincture did not appear to have much influence. There were eight cases of variola modificata. Twelve of the cases terminated fatally, one of which was of an hæmorrhagic type.

I need scarcely add, that I am fully convinced of the beneficial effects of tincture of iodine, not only as a powerful ectrotic remedy, but also as a very efficacious means of controlling the irritation and itching, and thereby not only relieving the suffering of the patient, but also removing the involuntary and irresistible disposition to scratch, and the consequent production of wheals and scars. I am also of opinion that it moderates the febrile action, and thereby the danger. I have used a small quantity of hydriodate of potass to aid in the solution of the iodine.

*I freely confess that I conceive I would not be doing justice and my duty to my patient, if I omitted to apply this remedy on any future occasion.* It should be commenced at the earliest day of the eruption, and continued daily for a week. I may add that I have been applied to, on many occasions, for iodine, by Indians from the Racquett River, to whom I could not afford further aid. The cases were generally of a very grave type, and it appears to me that the Indian constitution, like the Negro, suffers severely from this malady."

Besides the several medical gentlemen who saw these cases, during their progress, and subsequently after convalescense, I had the pleasure of showing such of the patients who had passed through the ordeal as I could meet with, to Dr. Marshall Hall, of London, during his visit to Montreal. Two of these (numbers 4 and 5) will be admitted not to have been selected as favorable specimens, not only from their severity, but also for one not having been seen by me till the 5th day, consequently not having had the application made till a late period; in fact these cases presented more marks than any other I had. Dr. M. Hall has kindly favored me with his opinion as follows:

*From Marshall Hall, M. D., F. R. S., &c.*

I have seen with much satisfaction several patients who had been afflicted by variola, and treated by Dr. Crawford by the application of the tincture of iodine. The superficial pits I noticed appeared to me to be so numerous and crowded, that confluence and deep and lengthened scars must have taken place, but for the effectual abortive treatment by the iodine; and I cannot but think this a most valuable application in such cases.

*Extract from a note from W. Henry, Esq., M. D., Inspector General of Military Hospitals.*

Since I received your communication on the use of tr. iodine as an ectrotic in small pox, I have directed it to be used, and careful minutes taken, in about a dozen bad cases of small pox in Military Hospitals, several of which I watched myself. I also observed the practice last year in two of your patients in the Montreal General Hospital.

In some of the military cases, the tincture was employed, but in the greater number a liniment was used, composed of powdered iodine and olive oil, in the proportion of a drachm of the former to an ounce of oil. I prefer this to the tincture for external use, because it adheres to the skin better and is not so easily evaporated.

I entertain no doubt of the great value of iodine in this practice. It appears to check the deepening and developing of the pustules, to prevent their confluence, and to lessen materially the cutaneous inflammation in the interpustular spaces. Though last, not least, by stopping the deepening of the pustules it prevents subsequent disfigurement by pockmarks.

*Extract from a note from P. W. Maclagan, M. D., Surgeon, 20th Regiment.*

I have employed the tincture of iodine in four cases of small pox, one of them semi-confluent, the others confluent and hemorrhagic. One which you saw terminated fatally, but the poor man felt great relief from the application, and earnestly begged its repetition more than once.

The others are decidedly less deeply marked than might have been expected. Indeed, the superficial traces which remain will, I doubt not, disappear entirely. One of my patients complained a good deal of the smarting, for an hour or two after the iodine was applied; but the remainder made mention only of the smell of it—rather I suspect the irritation of the mucous membrane produced by the vapor.

*From George W. Campbell, A. M., M. D., Professor of Surgery, McGill College.*

Within the last two months I have tried iodine as an ectrotic in small pox, in the Montreal General Hospital, in four cases; two of them severe cases of confluent small pox, in which the face and eyelids, on the second day of the eruption, were greatly swollen, and entirely covered with incipient pustules. The tincture used was composed of a drachm of iodine to the ounce of alcohol, a few grains iodid. potasse being added to dissolve the iodine. The application was repeated once a day for four or five successive days. No suppuration occurred on the face, and when the mask, formed by the iodine scaled off, there was no pitting, and the face presented a marked contrast to the skin on the limbs and body being perfectly smooth and healed over, long before the scabs had separated in other parts. In neither of the above cases did the constitutional symptoms correspond with the severity of the eruption. There was no secondary fever and I have no doubt the disease was greatly modified by the ectrotic treatment. In conclusion I would remark, that I think the strong tincture of iodine employed more effectual and less painful than the ordinary tincture.

*From A. Hall, M. D., Professor of Materia Medica, McGill College.*

I have employed tincture of iodine freely both in private and Hospital practice, and from the general good results which I have witnessed following its timely application, I deem it an essential part of the treatment in that complaint. Of the various ectrotics which have been suggested, I consider it incomparably the best.

Shortly after you first suggested its use, I admitted into my wards at the Montreal General Hospital, a young woman, laboring under a severe attack of variola secreta. Doubting the efficacy of the tincture, but desirous of testing its value, I ordered its application to the left side of the face, neck and arms. On recovery, these parts presented scarcely the appearance of a cicatrix, while the collateral portions were severely marked by the disease. I regarded this as an unequivocal demonstration of the value of its practice, although I deeply regretted afterwards that my doubts had suggested such a mode of experiment.

I agree with the view which you have expressed that success depends on as early an application of the tincture as possible, and a steady repetition of it during the maturating period of the disease. In females I have extended the application of it, over the breast, as well as over the face, and I have been rarely disappointed in my expectations.

I regard the use of iodine as a decided improvement in the treatment of small pox; and I am happy to bear my testimony to its value, and to the obligation under which society is to you as its suggester.

